# Blood Culture Utilization, Contamination Rates, Antibiotic Use, and Patient Outcomes in Intensive Care Units at a Tertiary Care Hospital in Western India: A Prospective Observational Study

**DOI:** 10.7759/cureus.109882

**Published:** 2026-05-29

**Authors:** Nirav D Gondaliya, Sangita Rajdev, Summiaya A Mullan

**Affiliations:** 1 Microbiology, Government Medical College, Surat, IND; 2 Microbiology, Government Medical College and New Civil Hospital, Surat, IND

**Keywords:** antibiotic usage patterns, antimicrobial resistance, antimicrobial stewardship program, intensive care unit stay, sterile blood culture

## Abstract

Background and objective

Bloodstream infections present a severe clinical challenge in intensive care units (ICUs), making timely and accurate blood cultures (BCs) critical for diagnosis. However, pre-analytical errors frequently compromise results and drive unnecessary broad-spectrum antibiotic use. The objective of this study was to describe baseline BC utilization, contamination rates, antimicrobial resistance profiles, and patient outcomes across different ICUs at a tertiary care hospital.

Methods

A 12-month prospective observational study was conducted at a 1000-bedded tertiary hospital in Surat, India. BC utilization, contamination rates, pathogen profiles, and defined daily doses (DDDs) were analyzed for 1704 cultures. Clinical outcomes were followed for 142 culture-positive patients.

Results

Overall BC utilization was 62.3 per 1000 patient-days, peaking in the medical ICU (MICU; 107.12). Observed high utilization units also show a high contamination rate, which was highest in the MICU (27%) compared to the neonatal ICU (NICU; 21%) and pediatric ICU (PICU; 19%). Gram-negative bacteria predominated (>79% across wards), with *Acinetobacter baumannii* (33.8%) and *Klebsiella pneumoniae* (30.3%) as leading isolates. Meropenem showed the highest consumption (4.55 DDD/100 patient-days). Mortality was significantly higher in the NICU (63.3%) compared to the MICU (30.8%) and PICU (25%) (p = 0.000).

Conclusions

High BC utilization in adult ICUs is associated with excessive contamination rates, highlighting a gap in diagnostic stewardship. The high prevalence of multidrug-resistant organisms and significant neonatal mortality underscores the need for standardized collection bundles and unit-specific antimicrobial policies to improve clinical outcomes.

## Introduction

Bloodstream infections (BSIs) are a leading cause of morbidity in intensive care units (ICUs). While blood cultures (BCs) are fundamental for diagnosis, high and indiscriminate use, particularly in adult ICUs, is often associated with increased contamination rates rather than improved pathogen detection. Contamination leads to false positives, unnecessary antibiotic exposure, and increased healthcare costs [1]. Before targeted quality improvement (QI) interventions can be successfully implemented, institutions must first accurately map their existing operational landscape. Therefore, the primary objective of this prospective observational study was to evaluate the baseline epidemiological parameters of diagnostic stewardship, specifically BC utilization, contamination rates, and their association with patient outcomes, across adult ICUs, neonatal ICUs (NICUs), and pediatric ICUs (PICUs). The secondary objective was to quantify targeted antibiotic consumption using the defined daily dose (DDD) methodology among the cohort of culture-positive patients, thereby establishing a baseline to inform future hospital-specific stewardship interventions.

## Materials and methods

Study area, design, and size

A 12-month prospective observational study was conducted to evaluate baseline BC utilization, contamination rates, and subsequent antibiotic consumption. The study took place across the medical ICU (MICU), NICU, and PICU of Government Medical College (GMC) and New Civil Hospital, a tertiary care teaching facility in Surat, India, from October 2024 to September 2025.

Study population

The study population comprised all adult, pediatric, and neonatal patients admitted to the participating ICUs who required BC investigations during the study period.

Inclusion and exclusion criteria

All patients admitted to the MICU, NICU, and PICU for whom a BC was clinically indicated and subsequently collected within the ICU setting were included. Patients whose BCs were drawn prior to ICU admission (e.g., in the emergency department, operating theaters, or general wards) were strictly excluded to isolate ICU-specific pre-analytical variables and outcomes. Fungal isolates were also excluded.

Sample size

A purposive, consecutive sampling strategy was employed over the 12-month study period. All eligible ICU patients meeting the inclusion criteria were monitored. Within this broader cohort, a specific subset of 142 culture-positive patients was identified for targeted antibiotic consumption analysis.

Study procedure

The observational procedure involved the daily prospective tracking of clinical and laboratory data. This included monitoring physician BC orders, tracking laboratory processing data to identify contamination events, and auditing pharmacy dispensing records to accurately calculate targeted antibiotic consumption following positive culture results.

Data were collected using a self-prepared pro forma, including demographic details, clinical history, BC collection details, antibiotic details, and outcome details. BC utilization rate was calculated as the total number of BC sets drawn divided by the total number of ICU patient-days, expressed per 1,000 ICU patient-days. This was calculated for the overall ICU cohort as well as stratified by individual units (MICU, NICU, and PICU) [2]. The contamination rate was calculated as the number of BC sets yielding a contaminant (e.g., coagulase-negative *Staphylococci*, *Corynebacterium *spp., or *Bacillus *spp. lacking clinical correlation) divided by the total number of BC sets collected, expressed as a percentage. BC positivity and antimicrobial resistance category were also assessed [3].

Clinical outcomes, including average length of ICU stay, central line-associated BSI (CLABSI) rate (per 1000 central line-days), and mortality, were prospectively recorded [4]. The most commonly used antibiotic usage was assessed using days of therapy (DOT) and DDD, measured using the World Health Organization’s DDD methodology [5]. It was calculated exclusively for the cohort of culture-positive patients (n = 142), with the total DDDs consumed divided by the total number of patient-days within this specific group, expressed per 100 patient-days.

Microbiological processing and resistance categorization

Antimicrobial Susceptibility Testing (AST) and Resistance Categorization

AST was performed utilizing standard laboratory protocols, specifically the standard Kirby-Bauer disk diffusion method. The interpretation of minimum inhibitory concentrations and zone diameters was conducted strictly in accordance with the prevailing Clinical and Laboratory Standards Institute (CLSI) M100 [6] performance standards. Based on these established breakpoints, culture-positive isolates were stratified into specific resistance phenotypes to analyze targeted antibiotic consumption, including the following:

Extended-spectrum beta-lactamase producers: *Enterobacterales *demonstrating non-susceptibility to extended-spectrum cephalosporins (e.g., ceftriaxone, ceftazidime, and cefotaxime) according to CLSI screening and confirmatory criteria.

Carbapenem-resistant organisms: Gram-negative bacilli (including *Enterobacterales*,* Pseudomonas aeruginosa*, and *Acinetobacter *species) demonstrating resistance to at least one carbapenem agent (imipenem, meropenem, or doripenem).

Methicillin-resistant *Staphylococcus aureus*: Defined by phenotypic resistance to cefoxitin or oxacillin.

Operational definitions

To ensure standardized reporting and replicability, the following operational definitions were applied throughout the study:

BC Contamination

A BC set was classified as “contaminated” if it yielded growth of common skin commensals (e.g., coagulase-negative *Staphylococci*, *Corynebacterium *species, *Bacillus *species other than *Bacillus anthracis*, *Micrococcus *species, or *Cutibacterium *species) in only one of a series of BC sets and the attending physician determined the isolate lacked clinical correlation with a true BSI, in accordance with CLSI M47 guidelines.

Multidrug-Resistant Organisms (MDROs)

Multidrug-resistant (MDR) was defined strictly according to the international expert consensus criteria, which classify an isolate as MDR if it demonstrates acquired non-susceptibility to at least one agent in three or more different antimicrobial categories [7].

CLABSI

CLABSIs were identified using the Centers for Disease Control and Prevention/National Healthcare Safety Network surveillance criteria. It was defined as a laboratory-confirmed BSI in a patient who had a central line in place for more than two consecutive calendar days on the date of the positive BC and where the infection was not related to an infection at another site [8].

Statistical analysis

Data were compiled using Microsoft Excel (Microsoft Corporation, Redmond, WA, USA) and statistically analyzed utilizing IBM SPSS Statistics for Windows, version 26.0 (released 2018; IBM Corp., Armonk, NY, USA). Categorical variables, including mortality rates, contamination rates, and pathogen frequencies, were expressed as frequencies and percentages. Differences in these categorical outcomes between the ICUs were evaluated using Pearson’s Chi-square test or Fisher’s exact test, as appropriate. To quantify the magnitude of association for primary outcomes, specifically, mortality differences between MDR and non-MDR infections, ORs and 95% CIs were calculated utilizing univariable logistic regression. Essential assumptions for logistic regression, including the independence of observations and adequate expected cell frequencies, were verified prior to interpretation. A two-tailed p-value of <0.05 was considered statistically significant.

## Results

**Figure 1:**
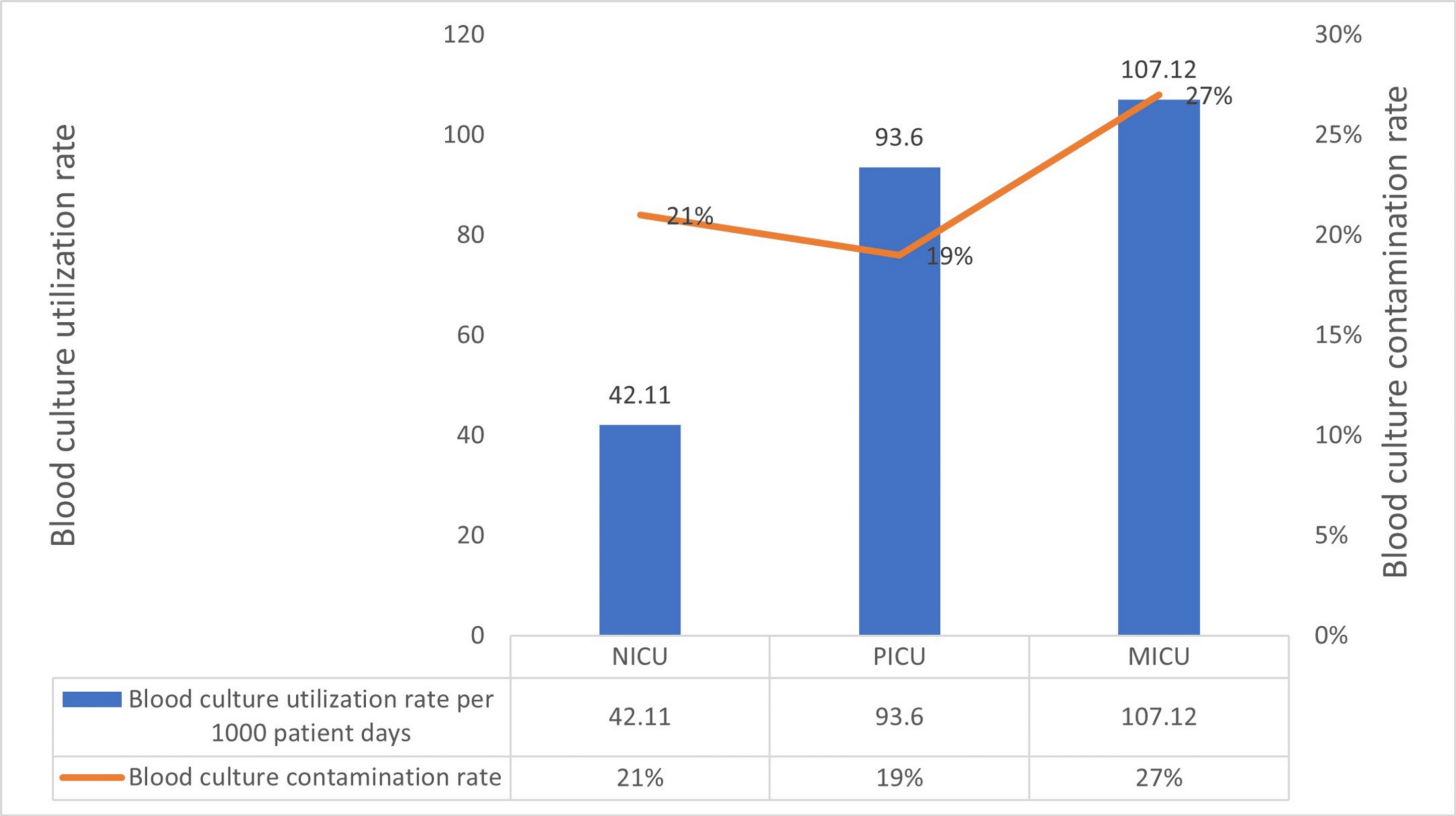
BC utilization rate (per 1000 patient-days) and contamination rate (%) from different ICU settings BC, blood culture; ICU, intensive care unit; MICU, medical intensive care unit; NICU, neonatal intensive care unit; PICU, pediatric intensive care unit

A total of 1,704 BCs were received during the study period. A total of 142 (10.2%) positive samples were identified. Figure 1 depicts BC utilization rates (per 1,000 patient-days) and contamination rates (%). Overall BC utilization was 62.3 per 1000 patient-days, with the highest rate in the MICU (107.12 per 1000 patient-days) and the lowest in the NICU (42.1 per 1000 patient-days). Overall contamination was n = 378/1704 (22.2%), and these rates varied significantly depending on the unit, peaking in the MICU at n = 112/412 (27.2%), compared to the NICU at n = 161/741 (21%) and PICU at n = 105/551 (19%) (Chi-square value (χ²) = 9.16, p = 0.010). Descriptively, it was observed that ICUs with higher BC utilization rates also recorded higher proportions of sample contamination.

Table 1 presents the microbiological profile, where Gram-negative bacteria predominated across all units. The most common isolates from positive BCs were *Acinetobacter baumannii *(48; 33.8%) and *Klebsiella pneumoniae *(43; 30.3%).

**Table 1:** Frequency of organisms from BC-positive patients in ICUs (N = 142) BC, blood culture; ICU, intensive care unit

Organism	No. of isolates (%)
Acinetobacter baumannii	48 (33.8%)
Klebsiella pneumoniae	43 (30.3%)
Gram negative bacilli (not identified)	10 (7%)
*Pseudomonas* spp.	10 (7%)
Staphylococcus aureus	9 (6.3%)
Escherichia coli	7 (4.9%)
*Enterococcus* spp.	7 (4.9%)
*Salmonella *Typhi	4 (2.8%)
Klebsiella oxytoca	2 (1.4%)
*Citrobacter *spp.	1 (0.7%)
Klebsiella aerogenes	1 (0.7%)

Of the 142 culture-positive patients, 68 (47.9%) were identified as having an MDR infection. For this epidemiological analysis, the individual patient (n = 142) was utilized as the primary denominator to calculate the MDR percentage. In cases of polymicrobial infections yielding multiple distinct isolates from a single patient, the patient was categorized overall as “MDR-positive” if at least one of the recovered pathogens met the MDR criteria. As previously defined, an isolate was classified as MDR if it demonstrated in vitro non-susceptibility to at least one agent in three or more distinct antimicrobial categories (Table 2).

**Table 2:** Analysis of MDROs among BC-positive patients across ICUs (N = 68) BC, blood culture; ICU, intensive care unit; MDR, multidrug resistant; MDROs, multidrug-resistant organisms; MICU, medical intensive care unit; NICU, neonatal intensive care unit; PICU, pediatric intensive care unit

Organism type	MICU (n = 26)	NICU (n = 33)	PICU (n = 9)	Total no. of cases (%)
MDR *Acinetobacter baumannii*	15	16	6	37 (54.4%)
MDR *Klebsiella* spp.	3	16	2	21 (30.9%)
Vancomycin-resistant* Enterococcus*	2	1	0	3 (4.4%)
MDR* Escherichia coli*	3	0	1	4 (5.9%)
MDR *Pseudomonas aeruginosa*	2	0	0	2 (2.9%)
MDR Gram-negative bacilli	1	0	0	1 (1.5%)

Table 3 summarizes the rate of antibiotic consumption, where meropenem showed the highest utilization, 4.55 DDD (per 100 patient-days), followed by amikacin, 1.64 DDD (per 100 patient-days). The average DOT was 18.2 days in the MICU, followed by 14.8 days in the PICU and 13.2 days in the NICU.

**Table 3:** Antibiotic usage calculated as DDD/100 patient-days across ICUs in BC-positive patients Data are represented as DDD per 100 patient-days. BC, blood culture; DDD, defined daily dose; ICU, intensive care unit; MDR, multidrug-resistant; MRSA, methicillin-resistant *Staphylococcus aureus*

Antibiotic	DDD/100 patient-days	Interpretation
Amikacin	1.64	Moderate use; used as empirical or targeted therapy for Gram-negative infections
Meropenem	4.55	Highest use; frequent use of broad-spectrum carbapenem for severe or resistant infections
Colistin	0.55	Very low use; reserved for MDR Gram-negative infections
Vancomycin	0.61	Low use; suggests limited MRSA coverage and selective use
Ceftriaxone	1.51	Moderate use

**Figure 2:**
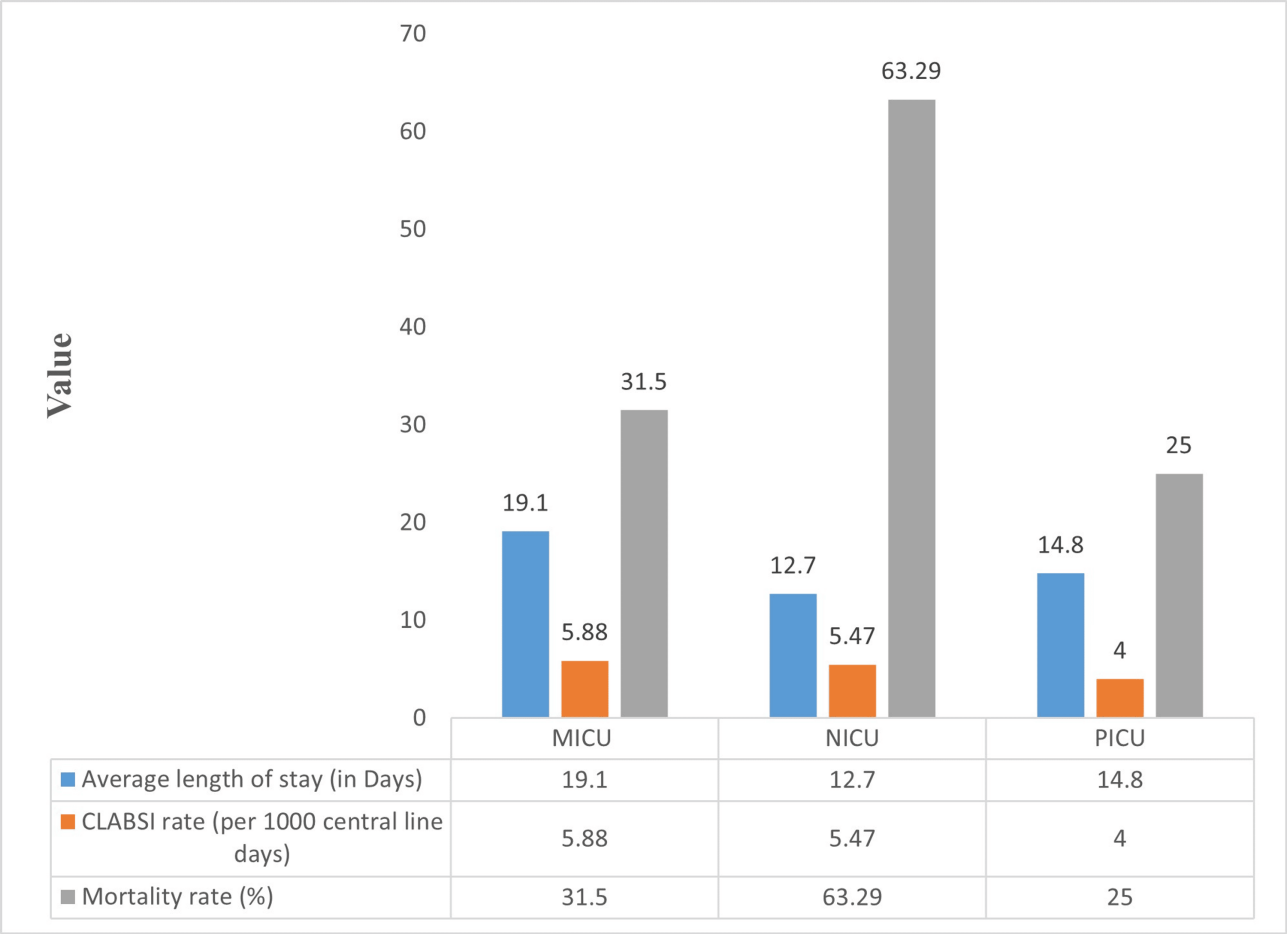
Distribution of patient clinical outcome indicators from positive BCs in each ICU Data are represented as means (length of stay), rates (CLABSI), and mortality (%). Statistical significance for mortality was determined using Pearson’s Chi-square test (χ² = 17.13, p < 0.001), where p < 0.05 was considered statistically significant. BC, blood culture; CLABSI, central line-associated bloodstream infection; ICU, intensive care unit; MICU, medical intensive care unit; NICU, neonatal intensive care unit; PICU, pediatric intensive care unit

Average length of stay was longest in the MICU, at 19.25 days, indicating higher acuity or prolonged recovery, while the NICU had the shortest stay, at 13.11 days. Mortality was significantly higher in the NICU, 50/79 (63.29%), compared to the MICU, 12/39 (30.76%), and the PICU, 6/24 (25.0%) (χ² = 17.13, p < 0.001). Furthermore, while clinical observations indicated a trend in which patients infected with MDROs (particularly *Klebsiella *spp. and *Acinetobacter*) faced higher odds of mortality compared to those with susceptible strains, this difference did not reach statistical significance (OR = 1.66, 95% CI: 0.85-3.21, p = 0.136). CLABSI rates were comparable across units, with the highest in the MICU, at 5.88 per 1000 central line-days, followed by the NICU, at 5.47 per 1000 central line-days, and the PICU, at 4 per 1000 central line-days. Overall, the NICU showed the highest patient load and mortality, whereas the PICU and MICU demonstrated similar infection trends, as shown in Figure 2.

## Discussion

BC utilization was highest in the MICU, followed by the PICU, and lowest in the NICU. Compared to Woods-Hill et al., PICU utilization was lower but comparable to post-intervention levels, indicating scope for further diagnostic stewardship, particularly in the MICU and PICU [1].

The overall contamination rate in our study was 22%, peaking at an alarming 27% in the MICU. This is substantially higher than the universally accepted CLSI benchmark target of <3% [9]. In resource-constrained tertiary care settings, several local systemic drivers likely contribute to this elevated pre-analytical error rate. These include a reliance on high-turnover rotating junior clinicians rather than dedicated, specialized phlebotomy teams; the frequent, pragmatic practice of drawing BCs through existing peripheral intravenous catheters rather than via fresh venipuncture; and potential nonadherence to optimal skin antisepsis contact times due to acute clinical workloads. To bridge this gap and align with CLSI targets, future institutional stewardship initiatives must transition from passive guidelines to concrete, structural interventions. Literature demonstrates that implementing dedicated BC phlebotomy teams, utilizing initial specimen diversion devices, adopting standardized sterile collection kits with chlorhexidine gluconate, and enforcing mandatory simulation-based training can successfully and sustainably drive contamination rates down to the ≤1-3% threshold in high-acuity environments. While studies by Hall et al. [10] and Self et al. [11] also reported elevated contamination in adult emergency and ICU settings, rates exceeding 20% severely undermine the predictive value of BCs and drive unnecessary carbapenem consumption. The stark contrast between our findings and international benchmarks highlights an urgent, critical need for the implementation of dedicated phlebotomy teams and standardized collection bundles in our setting.

*A. baumannii *was the predominant isolate in the MICU and PICU, while *Klebsiella *spp. predominated in the NICU, reflecting ward-specific differences in host factors and antibiotic pressure. These findings are consistent with studies by Edmond and Zaidi [12], Vincent et al. [13], and others, supporting the need for unit-specific infection control and empiric therapy policies.

Meropenem had the highest utilization among BC-positive patients, followed by amikacin, ceftriaxone, and levofloxacin. The frequent use of carbapenems and other broad-spectrum antibiotics reflects the predominance of Gram-negative infections and the need for early empiric therapy in critically ill patients. The use of reserve drugs like colistin and polymyxin B indicates the presence of MDROs. Similar antibiotic utilization patterns have been reported in other ICU studies, supporting the findings and highlighting the importance of antimicrobial stewardship [1,14].

Mortality was significantly associated with the ward of admission, with the NICU demonstrating a disproportionately high mortality rate (63.3%). This reflects extreme neonatal vulnerability, particularly to the predominant MDR *Klebsiella *spp. observed in this unit. Our findings align with Edmond and Zaidi [12], who highlighted the devastating impact of Gram-negative neonatal sepsis in resource-limited settings. However, our mortality rate is notably higher than the 33-35% mortality historically reported for BC-positive neonatal sepsis in India [15]. This stark increase suggests that the high prevalence of MDR strains (accounting for 85.3% of target infections) heavily restricted effective empiric therapeutic options, leading to adverse outcomes despite high meropenem utilization. No association with individual risk factors or CLABSI suggests multifactorial causes of mortality in ICU patients [12,16].

Strengths and limitations

A major strength of this study lies in its 12-month prospective observational design and the integration of microbiological profiles, antimicrobial utilization metrics, and patient outcomes within a unified epidemiological framework. Furthermore, the inclusion of diverse intensive care populations, spanning adult (MICU), neonatal (NICU), and pediatric (PICU) settings, provides a highly comprehensive, real-world picture of baseline BC practices and the localized MDR burden.

Our study has several important limitations that must be acknowledged. First, in addition to the single-center observational design and lack of longitudinal time-series tracking, no formal diagnostic stewardship intervention was implemented during the study period; therefore, the data represent baseline epidemiology rather than interventional outcomes. Second, due to data collection constraints, we were unable to incorporate standardized severity-of-illness scores (such as APACHE II or SOFA) or assess the precise timing and appropriateness of the initial empirical antibiotic therapies administered. Finally, our primary outcome and antibiotic consumption analyses were explicitly restricted to the subset of culture-positive patients (n = 142). While this allowed for a focused evaluation of definitive targeted therapy, it inherently limits the ability to draw broad inferences regarding the overall impact of pre-analytical stewardship on the wider culture-negative ICU population.

## Conclusions

The study demonstrates that while BCs are indispensable, “more is not always better.” In the MICU, high utilization rates did not necessarily lead to better pathogen yield but significantly increased the contamination burden to 27%. Furthermore, the alarming 63.3% mortality in the NICU associated with MDR *Klebsiella *spp. and *Acinetobacter *highlights a critical public health challenge. Effective diagnostic stewardship must involve both reducing unnecessary testing and strictly adhering to aseptic pre-analytical protocols to curb the cycle of false positives and subsequent carbapenem overreliance. Ultimately, this single-center observational study provides the foundational baseline data required to design and implement future targeted diagnostic stewardship QI initiatives aimed at minimizing sample contamination, optimizing antibiotic utilization, and improving critical patient outcomes.

## References

[1] Woods-Hill CZ, Colantuoni EA, Koontz DW (2022). Association of diagnostic stewardship for blood cultures in critically ill children with culture rates, antibiotic use, and patient outcomes: results of the Bright STAR collaborative. JAMA Pediatr.

[2] Saleh L, Chamieh A, El Basst R, Azar E (2025). The trends of blood culture contamination and utilization rates in an LMIC tertiary care center from 2010 to 2022: a call for diagnostic stewardship?. Antimicrob Steward Healthc Epidemiol.

[3] Chakraborty M, Sardar S, De R (2023). Current trends in antimicrobial resistance patterns in bacterial pathogens among adult and pediatric patients in the intensive care unit in a tertiary care hospital in Kolkata, India. Antibiotics (Basel).

[4] Hussain K, Khan MF, Ambreen G, Raza SS, Irfan S, Habib K, Zafar H (2020). An antibiotic stewardship program in a surgical ICU of a resource-limited country: financial impact with improved clinical outcomes. J Pharm Policy Pract.

[5] Kasabova S, Hartmann M, Werner N, Käsbohrer A, Kreienbrock L (2019). Used daily dose vs. defined daily dose—contrasting two different methods to measure antibiotic consumption at the farm level. Front Vet Sci.

[6] CLSI M100: performance standards for antimicrobial susceptibility testing. https://clsi.org/shop/standards/m100/.

[7] Magiorakos AP, Srinivasan A, Carey RB (2012). Multidrug-resistant, extensively drug-resistant and pandrug-resistant bacteria: an international expert proposal for interim standard definitions for acquired resistance. Clin Microbiol Infect.

[8] Centers for Disease Control and Prevention (CDC (2026). Bloodstream infection event (central line-associated bloodstream infection and non-central line associated bloodstream infection). Bloodstream Infection Event (Central Line-Associated Bloodstream Infection and Non-central Line-Associated Bloodstream Infection). National Healthcare Safety Network (NHSN.

[9] Fabre V, Hsu Y-J, Carroll KC (2025). Multicenter evaluation of blood culture contamination and blood cultures practices in US acute care hospitals: time for standardization. J Clin Microbiol.

[10] Hall RT, Domenico HJ, Self WH, Hain PD (2013). Reducing the blood culture contamination rate in a pediatric emergency department and subsequent cost savings. Pediatrics.

[11] Self WH, Speroff T, McNaughton CD (2012). Blood culture collection through peripheral intravenous catheters increases the risk of specimen contamination among adult emergency department patients. Infect Control Hosp Epidemiol.

[12] Edmond K, Zaidi A (2010). New approaches to preventing, diagnosing, and treating neonatal sepsis. PLoS Med.

[13] Vincent JL, Rello J, Marshall J (2009). International study of the prevalence and outcomes of infection in intensive care units. JAMA.

[14] Singh V, Singh NP, Nirmal K (2023). Occurrence of ESKAPE blood culture pathogens isolated in the intensive care units of a tertiary care teaching hospital in Delhi: a descriptive cross-sectional analysis. Int J Curr Microbiol App Sci.

[15] Adhikari NK, Fowler RA, Bhagwanjee S, Rubenfeld GD (2010). Critical care and the global burden of critical illness in adults. Lancet.

[16] Panda SK, Nayak MK, Jena P, Rath S, Gudu R, Pugulia R, Panda SS (2022). Nonfermenting, gram-negative bacilli causing neonatal sepsis in Odisha, India: four-year surveillance. Cureus.

